# Interaction of dietary fat intake with *APOA2, APOA5* and *LEPR* polymorphisms and its relationship with obesity and dyslipidemia in young subjects

**DOI:** 10.1186/s12944-015-0112-4

**Published:** 2015-09-13

**Authors:** Teresa Domínguez-Reyes, Constanza C. Astudillo-López, Lorenzo Salgado-Goytia, José F. Muñoz-Valle, Aralia B. Salgado-Bernabé, Iris P. Guzmán-Guzmán, Natividad Castro-Alarcón, Ma. E. Moreno-Godínez, Isela Parra-Rojas

**Affiliations:** Unidad Académica de Ciencias Químico Biológicas, Universidad Autónoma de Guerrero, Chilpancingo, Guerrero Mexico; Departamento de Biología Molecular y Genómica, Instituto de Investigación en Ciencias Biomédicas, Centro Universitario de Ciencias de la Salud, Universidad de Guadalajara, Jalisco, Mexico

**Keywords:** Dyslipidemia, Obesity, *LEPR*, *APOA2*, *APOA5*, Gene-diet interaction

## Abstract

**Background:**

Diet is an important environmental factor that interacts with genes to modulate the likelihood of developing disorders in lipid metabolism and the relationship between diet and genes in the presence of other chronic diseases such as obesity. The objective of this study was to analyze the interaction of a high fat diet with the *APOA2* (rs3813627 and rs5082), *APOA5* (rs662799 and rs3135506) and *LEPR* (rs8179183 and rs1137101) polymorphisms and its relationship with obesity and dyslipidemia in young subjects.

**Methods:**

The study included 200 young subjects aged 18 to 25 years (100 normal-weight and 100 obese subjects). Dietary fat intake was measured using the frequency food consumption questionnaire. Genotyping of polymorphisms was performed by PCR-RFLP.

**Results:**

Individuals carrying the *APOA5* 56 G/G genotype with a high saturated fatty acid consumption (OR = 2.7, *p* = 0.006) and/or total fat (OR = 2.4, *p* = 0.018), associated with an increased risk of obesity. We also found that A/G + G/G genotypes of the 668 A/G polymorphism in the *LEPR* gene with an intake ≥12 g/d of saturated fatty acids, have 2.9 times higher risk of obesity (*p* = 0.002), 3.8 times higher risk of hypercholesterolemia (*p* = 0.002) and 2.4 times higher risk of hypertriglyceridemia (*p* = 0.02), than those with an intake <12 g/d of saturated fatty acids. Similarly, *LEPR* 668 A/G + G/G carriers with a high fat total intake had 3.0 times higher risk of obesity (*p* = 0.002) and 4.1 times higher risk of hypercholesterolemia (*p* = 0.001).

**Conclusion:**

Our results suggest that dietary fat intake modifies the effect of *APOA5* and *LEPR* polymorphisms on serum triglycerides, cholesterol levels and obesity in young subjects.

## Background

Genetic variants associated with common polygenic obesity and dyslipidemias have been reported; unfortunately, many of these associations have been difficult to replicate [1, 2]. One hypothesis that may account for this inconsistency is that unexamined factors may modulate the existence of gene-environment interactions [3, 4]. Diet is one of the most important environmental factors that interacts with the genome to modulate disease risk. A better understanding of these interactions has the potential to support disease prevention via modification of dietary recommendations [5].

In the field of nutritional genetics (nutrigenetics), researchers study gene—diet interactions in an effort to better understand factors mediating individual response to dietary interventions [6]. Increasing energy intake is a major contributor to the current obesity epidemic. Data from the World Health Organization/Food and Agriculture Organization (WHO/FAO) showed that energy intake increased worldwide in about 450 kcal/person/day, and more than 600 kcal/person/day in developing countries from the mid-1960s to the late 1990s [7]. However, there are many other factors such as age, sex, physical activity, alcohol and smoking as well as genetic factors that will help identify vulnerable populations or individuals that will benefit from a variety of more personalized and mechanistic-based dietary recommendations [8].

Among the genes involved in the development of obesity and/or dyslipidemia are *LEPR*, *APOA2* and *APOA5*. Mutation of the *LEPR* gene results in leptin insensitivity, hyperphagia, morbid obesity, including metabolic and endocrine abnormalities [9]. APOA5 (protein) is regarded as an important modulator in the metabolism of triglycerides and in plasma it is associated with chylomicrons, very low density and high density lipoproteins [10]. In association studies, polymorphic variants in the gene have been associated with increased risk of obesity and metabolic syndrome [11, 12]. APOA2 is the second most common protein in high-density lipoproteins due to its involvement in lipid metabolism, as well as in the regulation of food intake [13, 14].

The aim of this study was to determine the interaction of dietary fat intake with the *APOA2* (rs3813627 and rs5082)*, APOA5* (rs662799 and rs3135506) and *LEPR* (rs8179183 and rs1137101) polymorphisms and its relationship with obesity and dyslipidemia in young subjects.

## Results

### Anthropometric, biochemical and nutritional characteristics

The total population for this study included 200 young subjects, 116 (58 %) were women and 84 (42 %) men and no significant differences were observed for age and gender. General characteristics of all subjects by group are shown in Table 1. Obese subjects had significantly elevated clinical and biochemical measures and lower HDL-cholesterol levels (all *P* < 0.05) than normal-weight controls. The evaluation of the dietary nutritional intake showed that obese group had a higher calorie and macronutrient consumption expressed in grams/day (g/d) than the control group but no differences were observed in cholesterol consumption (mg/d) (Table 1).

**Table 1:** Clinical, biochemical and nutritional variables between groups

Variables		Total	Control group	Obese group	*P*
		*n* = 200	*n* = 100	*n* = 100	
Clinical					
Age (years)^a^		20.9 ± 1.9	20.7 ± 1.9	21.2 ± 1.8	0.05
Sex^b^	Women	116 (58)	65 (65)	51 (51)	0.05
	Men	84 (42)	35 (35)	49 (49)	
Weight (kg)^c^		70.7 (56.5–91.5)	56.5 (51.1–91.5)	91.5 (82.4–99.6)	<0.001
Height (cm)^a^		162.7 ± 9.1	160.9 ± 8.9	164.5 ± 9	0.005
BMI (kg/m^2^)^c^		27.4 (21.5–33.1)	21.5 (20.3–23)	33.1 (31.4–35.3)	<0.001
Waist circumference (cm)^c^		88.3 (78–106)	78 (74.1–82)	106 (100.4–110.3)	<0.001
Hip circumference (cm)^c^		101 (92–115)	92 (88.5–110)	115 (110–119.3)	<0.001
Fat mass (g)^c^		23.3 (10.3–32.9)	10.3 (7.6–14.3)	32.9 (28.8–38.7)	<0.001
Fat percentage (%)^c^		28.4 (18.4–37.4)	18.5 (13.9–24.9)	37.4 (32.1–42.3)	<0.001
SBP (mmHg)^a^		109.8 ± 12.8	102.8 ± 9.8	116.9 ± 11.4	<0.001
DBP (mmHg)^c^		69 (63.5–76)	66 (60–71.5)	73 (67.5–80)	<0.001
Biochemical					
Glucose (mg/dL)^c^		83 (76–89)	80 (74–85)	85.5 (79–93)	<0.001
Total cholesterol (mg/dL)^c^		159.5 (141.5–181.5)	149.5 (132–176)	167.5 (148.5–192)	0.002
LDL-C (mg/dL)^c^		108 (85.5–130.5)	95.5 (81.5–119)	114 (88.5–146)	<0.001
Triglycerides (mg/dL)^c^		96 (69.5–144.5)	78 (60–103)	121 (91–165.5)	<0.001
HDL-C (mg/dL)^c^		44 (37–52.5)	47 (38–55)	42.5 (36–47.5)	0.005
Nutritional					
Total energy (kcal/d)^c^		2424.5 (2064–2835)	2184.5 (1902.5–2478.5)	2659.5 (2342.5–3063.5)	<0.001
Carbohydrates (g/d)^c^		318 (272.8–382.5)	298.5 (256.5–331.5)	350.5 (301.5–434)	<0.001
Protein (g/d)^c^		103 (85–125.5)	94.5 (78–113.5)	117.6 (96–140)	<0.001
Total fat (g/d)^c^		79 (61.5–96.7)	69.5 (58–86.5)	88.5 (73.5–103)	<0.001
SFA (g/d)^c^		11 (8–15)	9.5 (6–13)	13 (10–18)	<0.001
MUFA (g/d)^c^		17 (13–23)	15 (10.5–20)	20.5 (15–27)	<0.001
PUFA (g/d)^c^		9 (6–12)	7 (5–9)	11 (8–15)	<0.001
Cholesterol (mg/d)^c^		263.5 (171–341)	263.5 (188–330)	264 (168–35)	0.69

*BMI* body mass index, *SBP* systolic blood pressure, *DBP* diastolic blood pressure, *LDL* low-density lipoprotein cholesterol, *HDL* high-density lipoprotein cholesterol, *SFA* saturated fatty acids, *MUFA* monounsaturated fatty acids, *PUFA* polyunsaturated fatty acids

^a^Data provided in Mean ± SD; Student t test

^b^Data provided in n and percentage; Chi-square test

^c^Data provided in Median (percentile 25–75); Mann Whitney test

### Genotypic and allele frequencies

All polymorphisms in this study were in Hardy Weinberg Equilibrium in the control group: (χ^2^ = 0.01, *P* = 0.91 668A/G and χ^2^ = 2.6, *P* = 0.10 1968G/C in *LEPR* gene; χ^2^ = 0.27, *P* = 0.60 -1131 T/C and χ^2^ = 0.67, *P* = 0.41 56 C/G in *APOA5* gene, and χ^2^ = 1.35, *P* = 0.24 -265 T/C in *APOA2* gene). The *APOA2 -*1730 G/T polymorphism was monomorphic for the wild allele. No significant differences in the frequencies of genotypes or alleles between groups were observed (Table 2).

**Table 2:** Genotypic and allele frequencies between groups

Gene/polymorphisms	Genotype	Cases	Controls	*P*
	*n* (%)	*n* = 100	*n* = 100	
*LEPR*				
rs1137101				
A/A	63 (31.5)	33 (33)	30 (30)	
A/G	96 (48)	46 (46)	50 (50)	0.84^c^
G/G	41 (20.5)	21 (21)	20 (20)	
Allele				
A	222 (0.56)	112 (0.56)	110 (0.55)	0.84^b^
G	178 (0.44)	88 (0.44)	90 (0.45)	
rs8179183				
G/G	137 (68.5)	65 (65)	72 (72)	
G/C	59 (29.5)	31 (31)	28 (28)	0.10^a^
C/C	4 (2)	4 (4)	0 (0)	
Allele				
G	333 (0.83)	161 (0.80)	172 (0.86)	0.14^b^
C	67 (0.17)	39 (0.20)	28 (0.14)	
*APOA5*				
rs662799				
T/T	139 (69.5)	70 (70)	69 (69)	
T/C	59 (29.5)	30 (30)	29 (29)	0.60^a^
C/C	2 (1)	0 (0)	2 (2)	
Allele				
T	337 (0.84)	170 (0.85)	167 (0.84)	0.68^b^
C	63 (0.16)	30 (0.15)	33 (0.16)	
rs3135506				
C/C	11 (5.5)	4 (4)	7 (7)	
C/G	72 (36)	39 (39)	33 (33)	0.53^a^
G/G	117 (58.5)	57 (57)	60 (60)	
Allele				
C	94 (0.24)	47 (0.24)	47 (0.24)	1.0^b^
G	306 (0.76)	153 (0.76)	153 (0.76)	
*APOA2*				
rs5082				
T/T	143 (71.5)	74 (74)	69 (69)	0.56^a^
T/C	54 (27)	24 (24)	30 (30)	
C/C	3 (1.5)	2 (2)	1 (1)	
Allele				
T	340 (0.85)	172 (0.86)	168 (0.84)	0.57^b^
C	60 (0.15)	28 (0.14)	32 (0.16)	
rs3813627				
G/G	200 (100)	100	100	
G/T	-	-	-	-
T/T	-	-	-	
Allele				
G	400 (1.0)	200 (1.0)	200 (1.0)	-
T	-	-	-	

The genotypes analyzed in the population were in Hardy Weinberg Equilibrium

^a^Fisher’s exact test

^b^χ^2^ test

### Clinical, biochemical and nutritional characteristics according to APOA2, APOA5 and LEPR polymorphisms

The analysis of the clinical, biochemical and nutritional characteristics by genotypes in all subjects showed that the *APOA2* -265 T/T genotype carriers had a higher intake of polyunsaturated fatty acids (*P* = 0.02) than the T/C + C/C carriers. Furthermore, we found that the carriers of the T/C + C/C genotypes for the *APOA5* -1131 T/C polymorphism had decreased levels of HDL-cholesterol (*P* = 0.02) and an increased intake of polyunsaturated fatty acids (*P* = 0.037) compared with the T/T genotype (Table 3).

**Table 3:** Clinical, biochemical and nutritional variables according to polymorphisms in *APOA5*, *LEPR* and *APOA2* genes

Variables	Polymorphism −1131 T/C in the gene *APOA5*		*P*	Polymorphism −265 T/C in the gene *APOA2*		*P*	Polymorphism 668 A/G in the gene *LEPR*		*P*
	T/T	T/C + C/C		T/T	T/C + C/C		A/A	A/G + G/G	
	*n* = 139 (69.5)	*n* = 61 (30.5)		*n* = 143 (71.5)	*n* = 57 (28.5)		*n* = 63 (31.5)	*n* = 137 (68.5)	
Clinical									
Age (years)^b^	21 (20–22)	20 (19–22)	0.47	21 (19–22)	21 (20–22)	0.98	21 (20–23)	21 (19–22)	0.72
Weight (kg)^b^	74.3 (55.5–91.8)	69.5 (58.4–88.8)	0.76	74.3 (56.9–93.2)	68.2 (55.3–89.2)	0.80	75.7 (56.1–93.4)	69.5 (56.9–90.4)	0.94
BMI (kg/m^2^)^b^	30 (21.3–33.2)	24.4 (22–32.2)	0.72	30 (21.5–33)	24.1 (21.4–33.1)	0.62	30.7 (21.3–33.2)	24.7 (21.7–33)	0.92
Waist (cm)^b^	89 (78–106)	87 (79–105.8)	0.85	91.5 (79–106)	86 (77–102.5)	0.29	90 (78–106.5)	88.2 (78–104)	0.88
SBP (mmHg)^a^	109.3 ± 12.7	111.1 ± 12.9	0.35	110 ± 12.5	109.4 ± 13.6	0.77	108.4 ± 11.4	110.5 ± 13.4	0.28
DBP (mmHg)^b^	69 (64–75)	69 (63–76)	0.89	69 (63–76)	70 (64–74)	0.99	69 (63–76)	70 (64–75)	0.49
Biochemical									
Glucose (mg/dL)^b^	84 (76–89)	82 (74–88)	0.34	83 (76–89)	83 (78–89)	0.54	84 (76–89)	83 (76–89)	0.70
Cholesterol (mg/dL)^b^	160 (143–185)	150 (136–177)	0.14	160 (141–182)	157 (142–179)	0.67	153 (142–171)	162 (141–191)	0.07
LDL-C (mg/dL)^b^	109 (87–132)	106 (84–125)	0.57	110 (83–130)	106 (88–131)	0.99	103 (75–119)	111 (88–138)	0.02
Triglycerides (mg/dL)	94 (69–144)	104 (74–145)	0.32	94 (70–151)	98 (63–121)	0.32	93 (63–129)	100 (73–148)	0.19
HDL-C (mg/dL)^b^	45 (38–54)	41 (35–48)	0.02	44 (37–51)	45 (37–53)	0.95	43 (37–51)	45 (37–53)	0.50
Nutritional									
Total energy (kcal/d)^b^	2440 (2049–2860)	2411 (2179–2724)	0.79	2427 (2054–2854)	2411 (2130–2822)	0.86	2452 (2130–2764)	2418 (2022–2854)	0.72
Carbohydrates (g/d)^b^	318 (270.8–390)	318 (276–370)	0.87	318 (277–380)	320 (264–391)	0.74	318 (278–380)	318 (270–391)	0.89
Protein (g/d)^b^	105 (84–127)	100 (85–118.2)	0.56	101 (83–126)	107 (89–125)	0.63	108 (86.9–123)	101 (83–126)	0.46
Total fat (g/d)^b^	81 (61–98)	77 (65–95)	0.58	78 (62–96.5)	84 (61–97)	0.98	80 (65–97)	79 (61–96.5)	0.69
SFA (g/d)^b^	11 (8–16)	11 (8–15)	0.45	11 (9–15)	10 (7–15)	0.27	11 (8–14)	11 (8–16)	0.97
MUFA (g/d)^b^	17 (13–24)	16 (12–21)	0.18	17 (13–23)	16 (12–23)	0.23	17 (13–23)	17 (13–23)	0.86
PUFA (g/d)^b^	8 (6–12)	10 (8–12)	0.037	9 (7–13)	8 (6–10)	0.02	9 (7–12)	9 (6–12)	0.61
Cholesterol (mg/d)^b^	258 (172–359)	273 (152–329)	0.91	275 (186–345)	245 (143–328)	0.26	261 (143–329)	267 (181–357)	0.56

*BMI* body mass index, *SBP* systolic blood pressure, *DBP* diastolic blood pressure, *LDL* low-density lipoprotein cholesterol, *HDL* high-density lipoprotein cholesterol, *SFA* saturated fatty acids, *MUFA* monounsaturated fatty acids, *PUFA* polyunsaturated fatty acids

^a^Data provided in Mean ± SD; Student t test

^b^Data provided in Median (percentile 25–75); Mann Whitney test

In addition, the *LEPR* 668 A/G + G/G genotypes showed increased serum levels of LDL-cholesterol (*P* = 0.02) than those with the A/A genotype (Table 3). Serum lipid levels and dietary nutritional intake were not associated with *APOA5* 56 C/G and *LEPR* 1968 G/C polymorphisms in all subjects (data not shown).

### Gene-diet interactions for obesity and/or dyslipidemia

To estimate the association between consumption of saturated fatty acids and/or lipids with the presence of obesity and dyslipidemia we used logistic regression models adjusted for age, gender and physical activity; the results are given in Table 4. We found that risk of obesity and dyslipidemia was higher in individuals who had an increased intake of saturated fatty acids ≥12 g/d (OR = 3.9, 95 % CI 2.0–7.6; *P* < 0.001) and/or total fat ≥83 g/d (OR = 3.8, 95 % CI 1.9–7.7; *P* < 0.001).

**Table 4:** Interaction between polymorphisms studied with saturated fat intake (≥12 g/d) and total fat (≥83 g/d)

Variables	Obesity	Cholesterol	Triglycerides	LDL-C	HDL-C
	IMC ≥ 30 kg/m^2^	≥200 mg/dL	≥150 mg/dL	≥100 mg/dL	≤40 mg/dL
	OR (95 % CI); *P* ^a^				
SFA ≥ 12 g/d	3.9 (2.0–7.6); <0.001	1.8 (0.8–4.1); 0.15	1.3 (0.7–2.7); 0.4	1.5 (0.8–2.7); 0.2	1.0 (0.6–1.8); 0.9
Total fat ≥ 83 g/d	3.8 (1.9–7.7); <0.001	1.7 (0.7–3.9); 0.2	1.0 (0.5–2.2); 0.9	1.3 (0.7–2.4); 0.3	0.9 (0.5–1.8); 0.8
*APOA5* gene					
Polymorphism −1131 T/C					
Genotype T/C + C/C	0.9 (0.5–1.7); 0.7	0.7 (0.3–1.7); 0.4	0.9 (0.4–2); 0.9	0.8 (0.4–1.4); 0.4	1.9 (1.0–3.6); 0.05
Interaction T/C + C/C – SFA (≥12 g/d)	1.4 (0.5–3.5); 0.5	0.5 (0.1–2.2); 0.3	1.4 (0.5–3.8); 0.5	1.0 (0.4–2.5); 0.9	2.1 (0.9–5.0); 0.09
Interaction T/C + C/C – Total fat (≥83 g/d)	0.9 (0.4–2.5); 0.9	0.5 (0.1–2.5); 0.4	1.2 (0.4–3.5); 0.7	1.0 (0.4–2.4); 1.0	1.9 (0.8–4.8); 0.13
Polymorphism 56 C/G					
Genotype G/G	0.97 (0.5–1.8); 0.9	0.8 (0.4–1.8); 0.6	0.6 (0.3–1.2); 0.1	0.7 (0.4–1.3); 0.2	0.7 (0.4–1.2); 0.2
Interaction G/G – SFA (≥12 g/d)	2.7 (1.3–5.6); 0.006	0.97 (0.4–2.4); 0.9	1.2 (0.6–2.6); 0.6	0.8 (0.45–1.6); 0.6	0.8 (0.4–1.6); 0.5
Interaction G/G – Total fat (≥83 g/d)	2.4 (1.2–4.9); 0.018	1.1 (0.4–2.6); 0.9	1.0 (0.5–2.3); 0.9	0.7 (0.4–1.4); 0.4	0.8 (0.4–1.5);0.5
*LEPR* gene					
Polymorphism 1968 G/C					
Genotype G/C + C/C	1.3 (0.6–2.5); 0.5	0.7 (0.3–1.8); 0.5	0.8 (0.4–1.8); 0.6	0.8 (0.4–1.4); 0.4	0.8 (0.4–1.5); 0.5
Interaction G/C + C/C – SFA (≥12 g/d)	2.2 (0.9–5.3); 0.07	0.5 (0.1–1.9); 0.3	0.9 (0.4–2.4); 0.9	1.2 (0.5–2.7); 0.6	0.7 (0.3–1.7); 0.5
Interaction G/C + C/C – Total fat (≥83 g/d)	2.5 (1.0–6.2); 0.05	0.9 (0.3–2.8); 0.8	0.8 (0.3–2.2); 0.7	1.6 (0.7–3.7); 0.3	0.6 (0.2–1.3); 0.2
Polymorphism 668 A/G					
Genotype A/G + G/G	1.1 (0.6–2.1); 0.7	9.4 (2.1–41.5); 0.003	1.8 (0.8–3.9); 0.2	1.5 (0.8–2.8); 0.2	0.9 (0.5–1.7); 0.7
Interaction A/G + G/G – SFA (≥12 g/d)	2.9 (1.5–5.8); 0.002	3.8 (1.6–8.5); 0.002	2.4 (1.2–4.9); 0.02	1.9 (1.0–3.6); 0.05	1.3 (0.7–2.4); 0.4
Interaction A/G + G/G – Total fat (≥83 g/d)	3.0 (1.5–6.2); 0.002	4.1 (1.8–9.6); 0.001	1.6 (0.8–3.4); 0.2	1.6 (0.8–3.2); 0.14	1.3 (0.7–2.5); 0.4
*APOA2* gene					
Polymorphism −265 T/C					
Genotype T/C + C/C	0.7 (0.4–1.4); 0.3	0.9 (0.4–2.4); 0.9	0.5 (0.2–1.2); 0.1	0.8 (0.4–1.5); 0.5	1.0 (0.5–1.9); 0.9
Interaction T/C + C/C – SFA (≥12 g/d)	2.2 (0.8–5.6); 0.1	1.2 (0.4–3.9); 0.7	0.4 (0.1–1.6); 0.2	1.6 (0.6–3.9); 0.3	1.4 (0.6–1.2); 0.4
Interaction T/C + C/C – Total fat (≥83 g/d)	1.3 (0.6–3.1); 0.5	0.6 (0.2–2.2); 0.5	0.3 (0.08–1.1); 0.07	0.6 (0.3–1.4); 0.3	1.4 (0.6–3.1); 0.4

*OR* odds ratio, *CI* confidence interval, *SFA* saturated fatty acids; Reference category is genotype with major and/or minor frequency with low intake total fat and/or SFA, *SFA* saturated fatty acids, *LDL-C* low-density lipoprotein cholesterol, *HDL-C* high-density lipoprotein cholesterol

^a^OR adjusted by sex, age and physical activity

Similarly, we found that for polymorphism 668 A/G of the *LEPR* gene, the G allele carriers had 2 times higher risk of total cholesterol levels ≥200 mg/dL (OR = 2.1, 95 % CI 1.15–3.8; *P* = 0.008, data not shown), but when the analysis was performed by genotypes, we found that the AG + GG carriers had a high risk of hypercholesterolemia (OR = 9.4, 95 % CI 2.1–41.5; *P* = 0.003, Table 4).

The risk of obesity and/or dyslipidemia associated with dietary fatty acid intake of subjects according to *APOA2*, *APOA5* and *LEPR* genes are presented in Table 4. Individuals carrying the *APOA5* 56 G/G genotype with a high saturated fatty acid consumption ≥12 g/d (OR = 2.7, 95 % CI 1.3–5.6; *P* = 0.006) and/or total fat ≥83 g/d (OR = 2.4, 95 % CI 1.2–4.9; *P* = 0.018) were associated with an increased risk of obesity.

This analysis revealed that A/G + G/G genotypes of the 668 A/G polymorphism in the *LEPR* gene with an intake ≥12 g/d of saturated fatty acids, have 2.9 times higher risk of obesity (*P* = 0.002), 3.8 times higher risk of cholesterol levels ≥200 mg/dL (*P* = 0.002) and 2.4 times higher risk of triglyceride levels ≥150 mg/dL (*P* = 0.02), than those with an intake <12 g/d of saturated fatty acids. Similarly, *LEPR* 668 A/G + G/G carriers with a high fat total intake had 3.0 times higher risk of obesity (*P* = 0.002) and 4.1 times higher risk of cholesterol levels ≥200 mg/dL (*P* = 0.001) than those with a low intake of total fat <83 g/d.

## Discussion

Obesity is characterized by an energy imbalance between calories consumed and expended also known to be associated with cardiovascular risk factors such as dyslipidemia [15]. Dietary intake is associated with multiple health outcomes and is one of the critical, potentially modifiable environmental exposures to consider in gene-environment studies related with obesity [16].

In this study we found a gene-diet interaction for the 668 A/G polymorphism in the *LEPR* gene and 56 C/G in the *APOA5* gene with a high intake of saturated fatty acids (SFA) and total fat was associated with obesity, hypercholesterolemia and hypertriglyceridemia.

Differences have been reported in dietary intake between normal-weight and obese subjects. Our results are consistent with previous findings, where obese subjects have a higher intake in total energy, carbohydrates, total fat and protein as well as saturated, monounsaturated and polyunsaturated fatty acids, but no significant differences in cholesterol intake were observed, which explains excess body weight as a result of the high consumption of energy from macronutrients and in addition, fatty acids induce excess lipid accumulation in obese subjects. Furthermore, the obese group had increased lipid levels and low HDL-cholesterol levels, which are traditional cardiovascular risk factors associated with the high consumption of total fat and augmented adiposity [17].

Since the obese group had a high intake of macronutrients and total fat, suggests that consuming a high-fat diet has not effect on the satiety. However, whether fatty acid saturation differentially modulates satiety or energy intake is unclear. Maljaars and colleagues conducted a study in which fat emulsions were infused into the small intestine of 15 healthy subjects and found that triacylglycerol composed of unsaturated fatty acids (C18:2 and C18:1) significantly increased satiety and cholecystokinin secretion compared with the control group, without altering food intake, whereas those composed of saturated fatty acids (C18:0) had no effect [18]. Animal studies have established that ad libitum access to a high-fat diet promotes hyperphagia and obesity and is associated with leptin and insulin resistance [19]. The lack of consistency between measures of satiety/appetite and fat intake does not provide clarity on the role of saturated fat intake in the modulation of the satiety.

The frequency of the G allele polymorphism 56 C/G was higher in our population unlike other populations [20, 21]. The importance of this finding relies upon the association with obesity and intake of fatty foods found in this study, suggesting its role as an important risk factor for developing obesity in our population due to its high frequency.

Regarding the polymorphism 668 A/G in the LEPR gene, the G allele was found in 44 % of our population, but our results demonstrate discordance with those published in other populations [22, 23], as well as for other variants analyzed in this study. For this finding, we must point out that Mexican population is a mixture of Amerindian and European ancestry with a small proportion of African ancestry, however, the distribution can vary even within each region, which can also interfere with frequencies found in different populations [24].

Although a number of genes are associated with either weight or obesity and dyslipidemias, few have been shown to interact with diet and contribute to obesity and comorbidities. In this study, the *APOA5* -1131 T/C + C/C genotypes were associated with decreased levels of HDL-cholesterol and a high intake of polyunsaturated fatty acids (PUFA). Apolipoprotein A5 is found almost exclusively in association with lipoproteins as HDL, VLDL and chylomicrons [25]. In addition, the −1131 T/C polymorphism has been associated with low levels of APOA5 and therefore we suggest that it may be also be related with low levels of HDL [26]. Moreover, we found no gene-diet interactions in triglyceride levels contrasting with reports in other populations [27, 28], this could be due to the low risk allele frequency found in our population (0.16).

A significant interaction between *APOA5* 56G allele and high consumption of total fat and saturated fatty acids (SFA) associated with obesity but not with lipid levels, was found in this study. The 56 C/G gene variant, results in a change of serine to tryptophan at codon 19. Apolipoproteins and other polypeptides are known to contain N-terminal export signal sequences. Indeed, computational analyses for APOA5 predicted a strong export consensus sequence with a likely export cleavage site between amino acids 23 and 24 [29]. The change of a serine to a tryptophan residue at position 19 could reduce the rate of APOA5 export from the liver. Van den Berg et al., showed that high-fat diet contributes to the development of obesity and insulin resistance (IR) in APOA5-knockout mice (APOA5−/−). They also found that peripheral and central APOA5 protein administration in wild-type mice significantly reduced their food intake compared with APOA5 −/− mice. They suggest that low-circulating levels of APOA5 protein seen in rodents and humans may suggest a satiety-related signaling function, in addition to an effect of APOA5 on triglyceride hydrolysis [30].

Our study also found that *LEPR* 668G allele carriers have an increased risk of hypercholesterolemia, also the interaction of allele with high-fat diet promotes hypercholesterolemia, hypertriglyceridemia and obesity. Leptin generates its central and peripheral effects by binding to its receptors on the cell surface and subsequently activating downstream signaling pathways. Through posttranscriptional alternative RNA splicing, several isoforms of leptin receptors with identical extracellular and transmembrane domains but variable intracellular domains are expressed in humans [31]. Leptin binds to the long form of leptin receptor (LEPR) of multiple neuronal populations activating the JAK2/STAT3 pathway to regulate the synthesis of different neuropeptides implicated in the control of food intake and energy balance [32]. It has also been found that mutations of the *LEPR* gene results in leptin insensitivity, hyperphagia, morbid obesity, as well as metabolic and endocrine abnormalities [33].

The 668 A/G polymorphism includes a change of A to G in the leptin receptor gene, which is located in exon 6 encoding the extracellular region of the gene at nucleotide position 668, and confers an amino acid change at codon 223 (Q223R) changing the charge from a neutral amino acid (glutamine) to a positive (arginine) affecting receptor binding to its ligand [34]. However, we must point out that this is not the only polymorphism in this region of the gene, which may act in combination with other polymorphisms and non-genetic factors such as exercise, thereby contributing to what is known as leptin resistance, and thus, increasing the obesity phenotype. Furthermore, we observed that the lipolysis stimulator receptor (LSR) is actively involved in the regulation of postprandial lipemia. Based on the results found by Stenger et al. where they propose that the presence of leptin is important to maintain optimal levels of protein LSR in order to ensure efficiency of lipid metabolism during the postprandial phase, this can be complementary to its action as a satiety factor. However if leptin signaling is defective in the liver, this may induce suboptimal levels of protein expression of the LSR, which may contribute to elevated postprandial lipemia. This promotes increased lipemia in peripheral tissues, mainly in adipose tissue. As a result, it been proposed that leptin modulates LSR which promotes lipid removal during the postprandial phase and can contribute to the dynamics of the distribution and utilization of lipids in different tissues [35].

Our study also found that *APOA2* -265 T/T carriers had a greater consumption of polyunsaturated fatty acids (PUFA) unlike with the genotype T/C + C/C, but another study in individuals with European ancestry and genotype C/C, had an increased BMI and a high intake of total energy [36], this difference could be attributed to the sample size used in the study, which was comprised of 514 men and 564 women and found a greater number of C allele carriers. The *APOA2* gene is a member of the superfamily of apolipoproteins which include gene encoding soluble proteins such as APOA1 and APOA4 that share its genome structure and several functions. APOA4 has been associated with the regulation of food intake, satiety signal acting as well as APOA2 may be involved in the regulation of food intake [37].

Nonetheless, we did not find interactions between APOA2 -265 T/C and APOA5 -1131 T/C and dietary fat intake associated with obesity or serum lipid levels compared to other studies in Mediterranean, Asian and American populations [38–40]. These differences are supported by various reasons: first, the minor allele frequencies were low in our population (1.5 % for −265 T/C and 1 % for −1131 T/C polymorphisms), due to the small sample size; second, subject characteristics such as ethnicity, health status and other environmental and genetic factors are different; finally, our population presented different dietary patterns compared to other populations. However, future studies involving larger sample sizes are needed to confirm these findings.

## Conclusions

Our results suggest that dietary fat intake modifies the effect of polymorphisms at the *APOA5* and *LEPR* genes on serum triglycerides, cholesterol levels and obesity in young subjects. This gene-nutrient interaction could help to explain in Mexican subjects the variations in the lipid profile in response to fat intake, however, further studies are needed to confirm these findings.

## Methods

### Participants and study design

A total of 200 unrelated young subjects were recruited, aged 18 to 25 years old, 100 normal-weight (BMI 18.5 to 24.9 kg/m^2^) and 100 with obesity (BMI ≥30 kg/m^2^). None of the individuals were in treatment or diet to lose weight and pregnant women were not included in the study. Informed written consent was obtained from all participants according to the Declaration of Helsinki. The study was approved by the Research Ethics Committee of the University of Guerrero (registration number 012/2013).

### Anthropometric and biochemical analysis

Body weight was determined in light clothes and without shoes using a body composition monitor (TFB-300 GS, Tanita Corporation of America Inc USA). Height was measured with a stadiometer (BM-214, Seca, Hamburg, Germany). From these measurements, the body mass index was calculated as weight in kilograms divided by height in meters squared (kg/m^2^). Body circumferences were measured using an anthropometric tape (Seca 203, Hamburg, Germany). Waist circumference was measured at the umbilicus level and the superior iliac crest; and hip circumference was measured at the maximum point below the waist, without compressing the skin. Blood pressure was measured in the sitting position with an automatic sphygmomanometer (Omron MX3 Plus) on the left arm after 10 min rest, and the reading was reported in mmHg.

A fasting blood sample was taken to measure glucose, total cholesterol, HDL-cholesterol, LDL-cholesterol and triglycerides levels. Biochemical analysis was performed with commercially available kits (Spinreact) in semi-automated equipment Spinlab.

### Dietary assessment

Information on food and nutrition was obtained by means of a food-frequency questionnaire (FFQ), which was employed for the analysis of dietary data from the 2006 and 2012 ENSANUT Surveys [41]. The person responsible for data collection was a research dietitian trained in the methodology. Interviews were conducted from Monday to Friday. The questionnaire allowed the assessment in the consumption of food groups, energy, macronutrients and micronutrients with Nutrimind software (Mexico). The software contains over 2000 foods and allows the entry of other foods that are not consumed frequently. These calculations of macronutrients are based on the source tables foods like Mexican Food System Equivalents 1st and 2nd Edition, USDA (United States), Argenfoods (Argentina), Peruvian Journal of Cardiology (July to December 2000) and the table of food composition from Chile.

### DNA extraction and genotyping

Genomic DNA was extracted from white blood cells by the Miller technique and the screening for the *APOA2* (−1730 G/T, rs3813627 and −265 T/C, rs5082)*, APOA5* (−1131 T/C, rs662799 and 56 C/G, rs3135506) and *LEPR* (1968 G/C, rs8179183 and 668 A/G, rs1137101) polymorphisms, were performed by polymerase chain reaction-restriction fragment length polymorphism (PCR-RFLP). Primers for the polymorphisms were designed and restriction enzymes were identified using the Primer 3[42] and NEBcutter software, respectively [43]. All PCRs were performed in a research thermal cycler, using *Taq* polymerase (Invitrogen Life Technologies). Characteristics of the studied polymorphisms are presented in Table 5.

**Table 5:** Sequence of the primers, enzymes and sizes of PCR-RFLP fragments

Polymorphisms	Primer sequence		Annealing temp. (°C)	Product size (bp)	Restriction enzyme/allele sizes	
GEN *LEPR*						
						*BstUI*
1968 G/C (rs8179183)	F	5′-AATCCAGCCTACACAGTTGT-3′	63	224	G/G	224
	R	5′-CTTCCAAAGTAAAGTGACATTTTTCGC-3′			G/C	224, 198, 26
					C/C	198, 26
						*MspI*
668 A/G (rs1137101)	F	5′-AAACTCAA CGACACTCTC CTT-3′	63	80	A/A	80
	R	5′-TGAACTGACATTAGAGGTGA-3′			A/G	80, 57, 23
					G/G	57, 23
GEN *APOA5*						
						*Sau96I*
56 C/G (rs3135506)	F	5′-CACAGAGGTTGAGGCAGCAGA-3′	60	335	C/C	151, 77, 40, 35, 31, 1
	R	5′-GGCTCTGGCTCTTCTTTCAGG-3′			C/G	186, 151, 77, 40, 35, 31, 1
					G/G	186, 77, 40, 31, 1
						*MseI*
−1131 T/C (rs662799)	F	5′-GGAGC TTGTGAACGTGTGTATGAGT-3′	58	187	T/T	157, 30
	R	5′-CCCCAGGAAC TGG AGC GAA ATT-3′			T/C	187, 157, 30
					C/C	187
GEN *APOA2*						
						*SmlI*
−265 T/C (rs5082)	F	5′-GATAAGGTTGAGAGATGAGATCT-3′	62	310	T/T	310
	R	5′-GTGAGGATAAACAAGTTGGAGAA-3′			T/C	310,167,143
					C/C	167,143
						*NheI*
−1730 G/T (rs3813627)	F	5′-GAATAGTTCTGCTAGCACTTAC-3′	64	162	G/G	151, 11
	R	5′-CAAATGAGTACTACTCATTAAG-3′			G/T	162, 151, 11
					T/T	162

For the identification of all polymorphisms, quality control measures including positive and negative controls as well as replicated samples (10 % at random) were employed.

### Statistical analysis

The information obtained was analyzed in STATA statistical package v.12.0 (College Station, TX). For the descriptive analysis we obtained means and standard deviations of symmetric quantitative variables and comparison between groups was performed using the student t test and/or ANOVA; for the non-symmetric quantitative variables we obtained median and percentiles 25 and 75 and the comparison between groups was performed using the Mann Whitney and/or Kruskal Wallis. Absolute and relative frequencies for qualitative variables were determined and the comparison between the study groups (normal-weight vs. obese subjects) was performed using the Chi square test statistic (X^2^). The genotype and allele frequencies for the analyzed polymorphisms were performed by direct counting and Hardy-Weinberg equilibrium in the control group was calculated. Dietary fat intake was analyzed as both continuous and categorical variables, dichotomized by the population mean as a cut-off point. Two categories of saturated fat intake (<12 and ≥12 g/d) and total fat intake (<83 and ≥83 g/d) were considered. Logistic regression models, adjusted and unadjusted, were constructed to assess the association between the SNPs of the *LEPR*, *APOA2* and *APOA5* genes with a high dietary fat intake and determination of OR for the presence of obesity and dyslipidemia. *P*-values <0.05 were considered statistically significant.
